# Oral fosfomycin for treatment of urinary tract infection: a retrospective cohort study

**DOI:** 10.1186/s12879-016-1888-1

**Published:** 2016-10-11

**Authors:** Philippa C. Matthews, Lucinda K. Barrett, Stephanie Warren, Nicole Stoesser, Mel Snelling, Matthew Scarborough, Nicola Jones

**Affiliations:** 1 Department of Infectious Diseases and Microbiology, Oxford University Hospitals NHS Foundation Trust, John Radcliffe Hospital, Headley Way, Headington, Oxford, OX3 9DU UK; 2 Nuffield Department of Medicine, University of Oxford, Peter Medawar Building for Pathogen Research, South Parks Road, Oxford, OX1 3SY UK; 3 Pharmacy Department, Oxford University Hospitals NHS Foundation Trust, John Radcliffe Hospital, Headley Way, Headington, Oxford, OX3 9DU UK

**Keywords:** Gram-negative, UTI, *Escherichia coli*, Uropathogens, Urosepsis, Fosfomycin, Antibacterials, Antibacterial resistance

## Abstract

**Background:**

Fosfomycin is increasingly called upon for the treatment of multi drug-resistant (MDR) organisms causing urinary tract infection (UTI). We reviewed oral fosfomycin use for UTI treatment in a large UK hospital. The primary goal was to audit our clinical practice against current national guidelines. Secondary aims were to identify factors associated with treatment failure, and to investigate the potential for using fosfomycin in patients with co-morbidities.

**Methods:**

We retrospectively studied 75 adult patients with UTI who received 151 episodes of treatment with fosfomycin from March 2013 to June 2015. We collected clinical data from our electronic patient record, and microbiology data pre- and post- fosfomycin treatment. We recorded additional data for patients receiving prolonged courses in order to make a preliminary assessment of safety and efficacy. We also reviewed >18,000 urinary tract isolates of *Escherichia coli* and *Klebsiella spp*. processed by our laboratory over the final year of our study period to determine the prevalence of fosfomycin resistance.

**Results:**

There was a significant increase in fosfomycin treatment episodes over the course of the study period. Co-morbidities were present in 71 % of patients. The majority had *E. coli* infection (69 %), of which 59 % were extended spectrum beta-lactamase (ESBL)-producers. *Klebsiella* infections were more likely than *E. coli* to fail treatment, and more likely to be reported as fosfomycin resistant in cases of relapse following treatment. There were no adverse events in five patients treated with prolonged fosfomycin. Among all urinary isolates collected over a year, fosfomycin resistance was documented in 1 % of *E. coli* vs. 19 % of *Klebsiella* spp. (*p* < 0.0001).

**Conclusions:**

We report an important role for oral fosfomycin for MDR UTI treatment in a UK hospital population, and based on the findings from this study, we present our own local guidelines for its use. We present preliminary data suggesting that fosfomycin is safe and effective for use in patients with complex comorbidities and over prolonged time periods, but may be less effective against *Klebsiella* than *E. coli*.

**Electronic supplementary material:**

The online version of this article (doi:10.1186/s12879-016-1888-1) contains supplementary material, which is available to authorized users.

## Background

Urinary tract infections (UTI) are common and account for a significant burden of hospital admissions and associated healthcare expenditure [1]. Treatment has become more challenging due to an ageing population, high rates of comorbid disease and polypharmacy, allergy or intolerance to antimicrobial drugs, a growing number of patients with underlying immunological or anatomical defects, and the increasing prevalence of multi-drug resistant pathogens [1, 2]. Re-evaluation of ‘neglected’ antibacterial drugs is one approach to tackling this complicated burden of disease [3, 4]. One such agent, fosfomycin, is being called back into play in the UK for treating UTI [5, 6].

Characteristics that make fosfomycin appealing for the treatment of UTI include rapid absorption after oral administration, concentration for excretion in urine, biofilm activity [7, 8], and its efficacy against many multi-drug resistant organisms, including extended spectrum beta-lactamase (ESBL) and AmpC-producing Enterobacteriaceae [9]. Oral fosfomycin is well tolerated and largely free of serious adverse effects [10, 11], with only 5 % of patients reporting side-effects, most commonly diarrhoea [12].

Until recently, the only preparation of fosfomycin available in the UK was unlicensed Monuril (fosfomycin trometamol) imported from Germany. However, a licensed product has now become available (Mercury Pharmaceuticals). National guidelines have been published by two UK agencies, NICE (National Institute for Health and Care Excellence) and PHE (Public Health England); fosfomycin is recommended for uncomplicated UTI (defined as no fever/flank pain) caused by ESBL-producing *E. coli* in adults, if the prescription is endorsed by a microbiologist [13–15].

Approaches to dosing vary: NICE guidelines suggest a single dose of 3 g in women and two 3 g doses (at an interval of 3 days) in men [14], but the UK product licence is for a single dose only and European guidelines produced by the European Association of Urology do not recommend fosfomycin for use in men at all [16]. Although the UK recommendation is restricted to uncomplicated lower UTI [3, 14, 17, 18], and the focus of existing guidance is for out-patient treatment, fosfomycin has also been used with some success in patients with risk factors for persistent or recurrent UTI [11, 12, 19].

Nitrofurantoin is a potential alternative treatment for UTI caused by MDR *E. coli* [13], but is not recommended in the third trimester of pregnancy, and should be used with caution in patients with significant renal impairment. Guidance on the estimated glomerular filtration rate (eGFR) threshold for use has varied, generally being set at 45 ml/min, but also sanctioned for use in certain circumstances down to 30 ml/min [20]. Pivmecillinam is another potential choice of oral agent, but susceptibility testing has only recently been introduced in our institution, and was not in routine use until after the time period described by this study.

We retrospectively studied the use of fosfomycin in our large teaching hospital by auditing local prescribing against existing guidelines [14, 16], with the primary aim of developing a clear picture of the context in which this agent is currently used, and identifying ways in which prescribing can be optimized. Secondly, having identified a small group of patients receiving prolonged or recurrent treatment, we scrutinized these cases to gain insights into special situations that are not covered by current guidance, but in which fosfomycin may have a useful role. Finally, within the constraints of a retrospective study, we sought to identify preliminary evidence for factors predictive of treatment failure, in order to inform ongoing research efforts and to guide treatment decisions.

## Methods

### Study design

We undertook a retrospective observational study of oral fosfomycin use for UTI. We included all episodes of oral fosfomycin use for the treatment of UTI in adults age ≥16 years, from March 2013 (when this agent was first added to our local formulary) through to the end of June 2015, irrespective of urine culture results.

### Characteristics of patient cohort

The study centre is a large tertiary referral teaching hospital in Oxfordshire, UK, with >1400 in-patient beds serving a population of 805,000 (http://www.ouh.nhs.uk). Our population of individuals with complex, recurrent, resistant or persistent UTI is inflated by a tertiary referral service for urology, a large renal dialysis unit, and a regional renal transplant unit.

This cohort represents a group of patients who were all deemed well enough to receive treatment with an oral antibacterial agent. Routine clinical practice would be to admit any patient unwell with signs of systemic sepsis or clinical suspicion of pyelonephritis for monitoring, imaging, and treatment with intravenous antimicrobial therapy (this is a clinical assessment based on the whole picture, but would hinge on features such as fever >38 °C, tachycardia, hypotension, loin or back pain, and raised inflammatory markers).

We identified fosfomycin treatment episodes retrospectively using electronic records held by pharmacy. Based on the complex and changing product licence of fosfomycin, as well as recommendations made by NICE guidelines [14], local policy is that all fosfomycin prescriptions should be authorized by the infectious diseases/microbiology team. During the period of this study, general practitioners were not able to access fosfomycin, so all prescriptions were generated by hospital clinicians. Our practice is to give oral fosfomycin as monotherapy for UTI treatment.

### Data collection methods

We collected data on patient demographics, co-morbid diagnoses and laboratory parameters from the hospital electronic patient record (EPR, Powerchart), and culture results from electronic microbiology records (Sunquest). We were unable to gain access to sufficient paper records to review in detail the clinical symptoms at the time of every treatment episode. However, in the five patients who each received >10 fosfomycin doses, we did obtain paper-based clinical notes as an additional source of information.

### Analysis of fosfomycin resistance

In order to develop an overview of fosfomycin resistance in our region, we also reviewed antibacterial susceptibility data from *E. coli* and *Klebsiella* spp. isolated from all urine cultures processed by our laboratory during the final twelve-month period of our study. These represent unselected samples submitted from both primary and secondary care settings. We focused our analysis on these two organisms as they collectively account for >80 % of UTIs treated with fosfomycin (see ‘Microbiology of UTI treated with fosfomycin’ in results).

### Standards for evaluation of prescribing

We used guidance published by Public Health England (PHE) [15] and NICE [14] as standards against which to evaluate our own prescribing.

### Laboratory methods

Antimicrobial susceptibilities for uropathogens were determined using the BD Phoenix Automated Microbiology System (Becton Dickinson, Franklin Lakes, New Jersey; NMIC-75 panel. For fosfomycin, this contains Glucose-6-Phosphate). BD Phoenix utilises an optimised colorimetric redox indicator to detect active growth of an organism in the presence of the antimicrobial. The organism to be tested is grown on a non-selective medium in appropriate conditions (37 °C in O_2_ for *E. coli* / *Klebsiella* spp.) for 16–18 h, before a 0.5 MacFarland suspension is prepared (BD AutoPrep). This suspension is inoculated into the appropriate antimicrobial susceptibility testing panel (gram negative NMIC-75) that contains microwells pre-lined with increasing concentrations of antimicrobial. The panel is incubated at 35 °C on the instrument for up to 16 h, and automatically read every 20 min for growth. The MIC (minimum inhibitory concentration) for each antimicrobial is then determined by the concentration at which the organism fails to grow. The breakpoint for fosfomycin susceptibility was ≤32 mg/L for oral treatment of uncomplicated UTI based on breakpoint data published both by the European Committee for Antimicrobial Susceptibility Testing (EUCAST), available on-line at www.eucast.org, and the British Society for Antimicrobial Chemotherapy (BSAC) Standing Committee on Susceptibility Testing, available on line at http://bsac.org.uk.

ESBL-production was detected by BD Phoenix. For *E. coli* and *Klebsiella pneumoniae*, ESBL-positivity is reported based on an ESBL test (a differential response between the inhibitory effect of 2nd/3rd generation cephalosporins used alone or in combination with clavulanic acid) and/or an ESBL phenotypic pattern (resistance to piperacillin in combination with resistance to any of the ESBL screening drugs, cefotaxime, ceftriaxone, ceftazidime, cefpodoxime or aztreonam in organisms that are carbapenem-susceptible).

Other oral antibacterial agents for which susceptibility was routinely tested include amoxicillin, co-amoxiclav (amoxicillin/clavulanate), cephalexin, trimethoprim, nitrofurantoin and ciprofloxacin. During the study period, our laboratory did not routinely test susceptibility to pivmecillinam.

### Definitions for treatment outcome

Due to the retrospective, observational approach, this study was not designed to provide robust assessment of clinical cure. However we set out to interrogate the microbiology dataset for any preliminary evidence of outcome following fosfomycin treatment.

We divided the potential laboratory outcomes into the following five endpoint categories:

(i) No follow-up sample available;
(ii) Sterile urine;
(iii) Isolation of an indistinguishable organism compared to pre-treatment cultures (may represent relapse);
(iv) Isolation of a different organism compared to pre-treatment cultures (may represent re-infection);
(v) Mixed growth (may represent either a contaminated sample, or relapse/re-infection).

We used these five endpoints to group patients into the following two broad outcome categories:

- ‘Microbiological cure’ was our stringent cure definition, classifying only individuals for whom there was a sterile urine sample following their treatment episode as being cured (endpoint (ii) from the list above).
- ‘Functional cure’ was a more relaxed definition. Those classified as ‘cured’ in this case included those with microbiological cure as defined above (endpoint (ii)), but was also expanded to include patients for whom there was no follow-up sample (endpoint (i), suggesting likely clinical cure).

We excluded analysis of those for whom repeat cultures showed either pure growth of a different organism from baseline (endpoint (iv)) or mixed growth (endpoint (v)). This approach was based on the rationale that in patients with a different organism at follow-up, re-infection is the most likely explanation. This scenario is not suggestive of failure of index treatment with fosfomycin (and is rather a marker of the nature of this cohort in which patients have complex underlying reasons for recurrence of UTI, that is not related to failure of the antibacterial agent). Mixed cultures are of uncertain significance, potentially representing contamination of the urine sample, or recurrent infection with more than one organism; neither of these outcomes can be regarded as robust evidence of fosfomycin failure.

### Data analysis

**Fig. 1:**
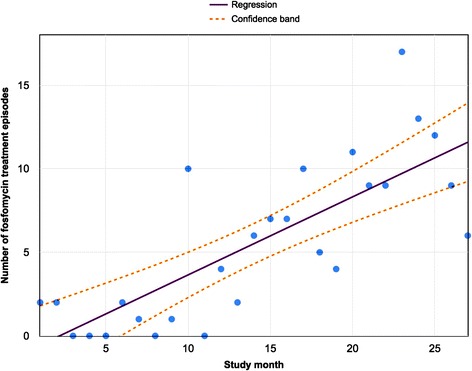
Number of fosfomycin treatment episodes by month in a cohort of adults treated for UTI in a UK teaching hospital. Each data point represents the number of unique fosfomycin prescriptions (irrespective of total dose) issued each month, from the time fosfomycin was first introduced to our formulary (March 2013) over 27 months to the end of the study period (May 2015). The solid line represents the trend computed by linear regression (slope = 0.46, 95 % CI 0.31-0.62, R^2^ = 0.6, *p* < 0.0001); the dashed lines indicate 95 % confidence intervals around the regression line

We recorded and analysed our data in a Microsoft Excel spreadsheet (Microsoft 2011; v14.5.7). Additional analysis was undertaken using Prism v6.0f, and on-line at http://graphpad.com. We used open access regression analysis tools in Google sheets (https://docs.google.com/spreadsheets) to produce Fig. 1.

For univariate analysis of factors associated with cure, we used the Mann–Whitney *U*-test to assess the statistical significance of differences between groups for continuous variables, and Chi-square/Fisher’s Exact Test for categorical variables (depending on sample size).

For multivariate analysis, we selected variables to enter based on those reaching statistical significance at univariate level (*Klebsiella* infections) and variables that we predicted *a priori* to predict fosfomycin treatment failure, namely in vitro fosfomycin resistance, creatinine clearance and comorbidity. Analysis was undertaken using a logistic regression approach in Google sheets (https://docs.google.com/spreadsheets).

### Study approval

This study was registered and approved by the Audit Department at Oxford University Hospitals NHS Foundation Trust. Informed consent was not deemed to be required.

## Results

### Characteristics of patient cohort

During the study period, 75 patients received oral fosfomycin, undergoing 151 treatment episodes (for raw data, see Additional file 1). There was a significant increase in fosfomycin prescriptions by month over the time period studied (Fig. 1). Females outnumbered males (57 vs. 18). The median age was 73 years (IQR 55–80; range 16–94), with no difference in age according to sex (*p* = 0.7, Mann Whitney *U* test).

Co-morbidities or risk factors for UTI were present in 52/75 patients (71 %), most commonly underlying renal tract disease (Table 1). eGFR was ≥10 mL / min in all patients. There was documented evidence of input from the microbiology/infectious diseases team in the EPR for 57/75 (76 %) index treatment episodes.

**Table 1:** Risk factors for urinary tract infection in a cohort of 75 adults treated with oral fosfomycin

Risk factor	Number of cases (% of all individuals treated)
GU tract pathology (stones, cancer of prostate/bladder/kidneys, urethral disease, self catheterisation)	25 (33.3)
Renal transplant	12 (16.0)
Systemic disease (non-renal tract malignancy, steroids, diabetes, cardiovascular disease, GI tract disease)	12 (16.0)
Pregnant	3 (4.0)
No documented risk factor(s) ^a^	23 (30.7)
TOTAL	75

^a^ No cases of recent urological intervention or patients with indwelling urethral catheters were identified on the basis of retrospective review of electronic records

### Microbiology of UTI treated with fosfomycin

We analysed microbiology data initially focusing just on the first treatment episode for each patient. The majority had urine cultures positive for *E. coli* (52/75, 69 %; Table 2). ESBL-production was reported in 31/52 of the *E. coli* isolates (59 %) and 6/9 *K. pneumoniae* (67 %); (Table 2). Overall, therefore, only 31/75 (41 %) of all isolates met the microbiological criterion (ESBL *E. coli*) for fosfomycin treatment stipulated by NICE guidance [14]. This result reflects a discrepancy between our own clinical practice and current guidelines.

**Table 2:** Urine culture results obtained at index episode of UTI in 75 adults treated with oral fosfomycin

Urine culture result	Number (% of all patients)^a^	Number with ESBL (% of cases with this organism)^b^	Number nitrofurantoin sensitive (% of cases with this organism)^b^	Number fosfomycin sensitive (% of cases with this organism)^b^
*Escherichia coli*	52/75 (69.3)	31/52 (58.5)	30/52 (56.6)	49/50 (98.0)
*Klebsiella pneumoniae*	9/75 (12.0)	6/9 (66.7)	1/9 (11.1)	9/9 (100.0)
Other Enterobacteriaceae in pure growth	6/75 (8.0)	0/6 (0)	0/6 (0)	5/6 (83.3)
Pseudomonas	1/75 (1.3)	n/a	n/a	n/d
Mixed growth	4/75 (5.3)	n/a	n/a	n/a
No significant growth	2/75 (2.7)	n/a	n/a	n/a
No data	1/75 (1.3)	n/a	n/a	n/a
Total	75/75	37/61	31/61	63/65

*n/a* not applicable, *n/d* no data

^a^The denominator in this column is the total number of patients with a urinary isolate (*n* = 75)

^b^The denominator in these columns is adjusted according to data availability / relevance. Note that fosfomycin susceptibility data were missing for two *E. coli* isolates

Unexpectedly, fosfomycin was prescribed for two patients with a urinary isolate that was reported as fosfomycin resistant in vitro; one of these was *E. coli* and the other *Enterobacter aerogenes*. There were a further two isolates for which an in vitro fosfomycin susceptibility was not reported (both *E. coli*).

### Treatment episodes and dose schedules

The majority of individuals (*n* = 53/75; 71 %) had just one fosfomycin treatment episode during the study period; eight patients had two treatment episodes and fourteen patients received ≥3 treatment courses. There was no relationship between age or sex and receipt of >1 treatment episode (*p* = 0.9 and *p* = 0.2, respectively).

NICE guidelines recommend a single dose of fosfomycin for females, and two doses (off-licence) for males [13, 15]. Among females in this study cohort, 45/57 (79 %) first treatment episodes adhered to the single dose recommendation [13, 15] with the remaining 12 being prescribed two or three doses. In contrast, among males, only 8/18 (44 %) received the recommended ≥2 doses. However, across all 151 treatment episodes, men were more likely to be prescribed additional fosfomycin doses (median 1 dose in women, versus median 3 doses in men *p* < 0.0001; data not shown).

### Potential for treatment with nitrofurantoin

Among index treatment episodes, 31/75 (41 %) patients had an isolate that was nitrofurantoin susceptible; 30/31 of these were *E. coli* (Table 2). Among the 31 patients with nitrofurantoin-susceptible isolates, 30 had contemporaneous measurement of renal function. Of these 30, four were ineligible for nitrofurantoin due to eGFR <30 mL/min, one was pregnant, and another had previously failed to respond to nitrofurantoin. This leaves a total of 24 individuals for whom there was no documented contra-indication to nitrofurantoin, but who received fosfomycin instead.

### Prolonged or recurrent fosfomycin treatment

In five individuals, treatment with standard short-course fosfomycin failed to sterilize the urinary tract, and longer courses were used (Table 3). There were no documented concerns about safety or tolerability, and infection was either cured or suppressed sufficiently to keep the patient out of hospital during the treatment course, although two subsequently relapsed and have been re-treated.

**Table 3:** Summary of five patients treated with more than ten 3 g doses of oral fosfomycin for UTI

Study number	Age group at first dose (years)	Gender	Clinical background, including possible risk factors for UTI	Urine culture result	Total number of 3 g fosfomycin doses	eGFR at baseline	Any reported side-effects	Notes on outcome
FOS015	80–89	M	Vasculopath; bilateral below knee amputations. Recurrent *E. coli* bacteraemia with unclear source despite extensive investigation. Relapsed after a treatment course of iv ertapenem	ESBL *E. coli*	36	79	None reported	Well when reviewed following end of treatment course; discharged from clinic
FOS038	60–69	M	History of recurrent UTI for > 10 years. Dilatation for urethral stricture, followed by TURP. Prostate cancer confirmed; treated post-op with radiotherapy	ESBL *E. coli*	81	>90	None reported	Interval free of infection, followed by recurrence of UTI (different organisms)
FOS060	60–69	M	Relapsing urosepsis following TRUS biopsy (negative for prostate cancer). Presumed prostatitis as focus of infection.	ESBL *E. coli*	33	79	Reported shortness of breath during treatment, but thought unlikely to be related to fosfomycin	Well when reviewed following end of treatment course; discharged from clinic
FOS098	70–79	M	Transitional cell carcinoma treated with nephrouretectomy. Regular surveillance cystoscopy complicated by recurrent UTI; recurred after completing treatment with meropenem.	ESBL *E. coli*	49	41	None reported	Interval free of infection, followed by recurrence and re-treatment with fosfomycin
FOS140	40–49	F	Recurrent UTI following sling procedure and botox treatment for stress/urge incontinence. Intermittent self-catheterisation. Good symptomatic relief with fosfomycin.	*E. coli*	15	>90	None reported	Ongoing prophylaxis while awaiting urogynaecology surgery

*eGFR* estimated glomerular filtration rate, *TURP* trans-urethral resection of the prostate, *TRUS* trans-rectal ultrasound guided prostate biopsy

### Treatment outcomes

Although this study was not specifically designed to ascertain outcomes, we sought preliminary evidence for the success of treatment, using two different definitions, ‘functional’ cure and ‘microbiological’ cure (see Methods). Follow-up culture results were obtained at a median of 13 days following the first fosfomycin dose (range 2–241 days; IQR 7–40 days).

Follow-up outcomes based on urine cultures were divided into five categories as follows: no follow-up sample available (22/75; 29.3 %), sterile urine at follow-up (21/75; 28 %), isolation of the same organism compared to pre-treatment cultures (19/75; 25.3 %), isolation of a different organism compared to pre-treatment cultures (8/75, 10.6 %) and mixed growth (5/75; 6.6 %).

(i) Microbiological cure: Using this stringent definition of cure, only 40/75 cases could be classified (the remaining 35 either had no post-treatment culture data (*n* = 22), had culture data more suggestive of reinfection (*n* = 8), or a mixed/contaminated sample (*n* = 5)). Of the 40 that could be classified (Table 4), 21 (53 %) met the criteria for cure, and no factor was statistically associated with failure.

**Table 4:** Univariate analysis of clinical and laboratory variables as predictors of outcome for fosfomycin treatment of urinary tract infection in adults. Cure and fail are classified according to ‘functional’ definition (results presented in data columns 1–3) or ‘microbiological’ definition (results presented in data columns 4–6)

Predictor	‘Cure’ (functional definition) Total *n* = 42	‘Fail’ (functional definition) Total *n* = 19	*p*-value (functional definition)	‘Cure’ (microbiological definition) Total *n* = 21	‘Fail’ (microbiological definition) Total *n* = 19	*p*-value (microbiological definition)
ESBL-positive	20/37	10/19	1.0^a^	12/21	10/19	1^a^
Nitrofurantoin resistant	17/37	12/19	0.3^a^	8/21	12/19	0.2^a^
Fosfomycin resistant	1/35	1/19	1.0^a^	0/20	1/19	0.49^a^
Male	14/42	4/19	0.4^a^	4/21	4/19	0.72^a^
*Klebsiella pneumoniae*	2/37	5/19	0.03^a^	2/21	5/19	0.22^a^
Comorbidity (any)	32/42	15/19	1.0^a^	12/21	15/19	0.19^a^
Transplant	8/42	3/19	1.0^a^	4/21	3/19	1^a^
Urethral catheter	4/42	3/19	0.66^a^	2/21	3/19	0.65^a^
Median age, years (IQR)	75 (62–81)	64 (42–79)	0.13^b^	70 (58–78)	64 (42–79)	0.47^b^
Median eGFR, ml/min (IQR)	70 (41–87)	>90 (29– > 90)	0.2^b^	64 (38–89)	>90 (29– > 90)	0.26^b^
Median CRP, mg/L (IQR)	21 (3–93)	25 (6–62)	1.0^b^	16 (2–78)	25 (6–62)	0.84^b^

Total number of patients represented is 61 for functional cure (‘cure’ group (*n* = 42) is patients with a sterile urine on follow-up culture, or no follow-up culture; ‘fail’ group (*n* = 19) is patients with recurrent growth of an indistinguishable organism). Total number of patients is 40 for microbiological cure (‘cure’ group (*n* = 21) is patients with a sterile urine on follow-up culture; ‘fail’ group (*n* = 19) is patients with recurrent growth of an indistinguishable organism)

The denominator in some rows is smaller than the overall total due to missing data, including no culture / susceptibility data and ESBL-status unknown or not applicable

*eGFR* estimated glomerular filtration rate, *CRP* C-reactive protein

^a^Fisher’s Exact Test; ^b^ Mann Whitney *U* test
(ii) Functional cure: Among 75 index treatment episodes, 61 patients could be classified using this more relaxed cure definition (the remaining 14 were equivocal due to post-treatment urine culture being unavailable (*n* = 1), growing a different organism (*n* = 8) or a mixed/contaminated sample (*n* = 5)). Of these, 42/61 (69 %) were considered cured. The only factor associated with failure on univariate analysis was infection with *K. pneumoniae* (*p* = 0.03; Table 4). This finding should be interpreted with caution as statistical significance is lost when correction for multiple comparisons is undertaken (e.g. by a Bonferroni approach). However, undertaking a multivariate approach to analysis again demonstrated *Klebsiella* as a significant predictor of treatment failure (*p* = 0.04; Table 5).

**Table 5:** Multivariate analysis of clinical and laboratory variables as predictors of outcome for fosfomycin treatment of urinary tract infection in adults. Cure and fail are classified according to ‘functional’ definition (results presented in data columns 1–3) or ‘microbiological’ definition (results presented in data columns 4–6)

Predictor	‘Cure’ (functional definition) Total *n* = 33	‘Fail’ (functional definition) Total *n* = 19	*p*-value (functional definition)	‘Cure’ (microbiological definition) Total *n* = 20	‘Fail’ (microbiological definition) Total *n* = 19	*p*-value (microbiological definition)
Fosfomycin resistant in vitro	1/33	1/19	0.21	0/20	1/19	0.99
*Klebsiella pneumoniae*	2/33	5/19	0.04	2/20	5/19	0.13
Comorbidity (any, including transplant/urethral catheter)	21/33	14/19	0.22	15/20	14/19	0.47
Median eGFR, ml/min (IQR)	64 (41–86)	90 (29–90)	0.19	64 (37–87)	90 (29–90)	0.20

Total number of patients represented is 52 for functional cure (‘cure’ group (*n* = 33) is patients with a sterile urine on follow-up culture, or no follow-up culture; ‘fail’ group (*n* = 19) is patients with recurrent growth of an indistinguishable organism)

Total number of patients represented is 39 for microbiological cure (‘cure’ group (*n* = 20) is patients with a sterile urine on follow-up culture; ‘fail’ group (*n* = 19) is patients with recurrent growth of an indistinguishable organism)

*eGFR* estimated glomerular filtration rate

### Selection of fosfomycin resistance within this cohort

On follow-up urine culture, there were 19 instances in which the identification of the organism was the same as that from pre-treatment culture. In five isolates that had been fosfomycin susceptible at baseline, the follow-up culture was reported fosfomycin resistant (four *Klebsiella pneumoniae* and one *Morganella morganii*). Selection of resistance was significantly more frequent in non-*E. coli* Enterobacteriaceae than in *E. coli* (5/6 vs. 0/13; *p* = 0.0005).

### Fosfomycin resistance patterns in all urine isolates of *E. coli* and *Klebsiella*

Of 18,474 urinary *E. coli* and *Klebsiella spp*. isolates processed by our laboratory in the final twelve months of the study, 511 were fosfomycin resistant (3 %). The majority of these were *Klebsiella* spp. (363/1888 (19 %) fosfomycin resistant, versus 148/16586 (1 %) for *E. coli*; *p* < 0.0001). There was no relationship between ESBL-production and fosfomycin resistance (data not shown).

### Mortality

Our electronic patient record did not record any patient deaths. However, this study was not designed to capture mortality data robustly and we cannot exclude the possibility that patients referred for treatment from outside our region may subsequently have died.

## Discussion

As the global prevalence of drug resistance increases, fosfomycin is likely to become increasingly called upon for the oral treatment of UTI [21], as well as for other infections of the urogenital tract including prostatitis [22]. This pattern is reflected in our own centre, in which we show that fosfomycin prescriptions have increased over time. Within the constraints of retrospective analysis, it is difficult to be certain what underpins this change, but we can postulate that the increase in treatment episodes is most likely to reflect increased clinician awareness of fosfomycin as a formulary agent, recognition of NICE guidelines, and potentially an increase in the number of patients presenting with MDR organisms over time.

Current NICE guidelines [14] are based on only four small studies (for summary, see Additional file 2) of which two were undertaken in Turkey, one in Spain and one in North America [10, 11, 19, 23]. This cohort is therefore a significant recent addition to the data, and offers a unique snapshot of a UK population. A median age of 73 is likely to reflect the increasing risk of UTI with age [24], as well as the fact that co-morbid problems increase with age.

In our subgroup analysis, only 21 of 40 cases (53 %) met our most stringent definition of cure. Notably, 22 cases were excluded from this analysis as there was no follow-up data; this may well artificially lower the proportion of cases that we were able to report as cured. Low cure rates may also be a result of the complex population described here (co-morbidity, older age, urethral catheters), but due to small numbers none of these factors was found to be independently predictive of treatment failure (Table 4).

We identified several notable patterns in fosfomycin prescribing and outcomes. Firstly, males were significantly less likely to receive the recommended fosfomycin treatment dose than females based on NICE guidance [13]. Secondly, our data suggest a lower chance of cure in UTI caused by *Klebsiella* spp., consistent with higher local in vitro resistance rates, also observed elsewhere [25–27]. Interestingly, however, the failure of fosfomycin in this cohort occurred even with *Klebsiella* isolates that were reported as fosfomycin susceptible pre-treatment. In the absence of robust prospective data collection, we can only speculate why fosfomycin might have been used in two patients with an isolate reported as resistant in vitro. The most likely explanations are that the fosfomycin was prescribed prior to the full susceptibility data being released from the laboratory and/or the patient had a previous urinary isolate that was reported as fosfomycin susceptible.

Thirdly, this cohort is of particular interest in being enriched for individuals with significant risk factors for recurrent, resistant and difficult-to-treat UTI due to the tertiary referral specialties in our centre. We did not find any association between co-morbidities and increased risk of treatment failure in this cohort. This is preliminary evidence that fosfomycin can be safe and effective for treating UTI even in this complex group, although larger numbers and prospective studies are needed to assess this with greater confidence.

Finally, in this cohort, nearly one-third of patients were potentially eligible for treatment with nitrofurantoin, which is licensed, easier to access, and cheaper [14]. However, the retrospective approach to data collection meant it was not possible to identify the proportion of these patients in whom there was a contra-indication to nitrofurantoin. Another alternative agent for the treatment of urinary tract infection caused by ESBL-producing Enterobacteriaceae is pivmecillinam [28], which may increasingly be called into play.

Concerns regarding wider use of fosfomycin include tolerability, cost, and the spread of resistance; we address these issues here in turn.

Regarding tolerability, in our five patients who received multiple fosfomycin doses, the clinical notes suggested the drug was well tolerated. This is consistent with previous reports [19, 22, 23, 29].

In terms of economic implications in the UK, a single sachet of fosfomycin 3 g can currently cost £10-60 depending on the supply route (unlicensed or licensed product) compared to an equivalent three day treatment regimen with nitrofurantoin for which the total is < £3 [14]. Although nitrofurantoin is cheaper, there are potential disadvantages, including uncommon (but potentially serious) side effects, and the need for longer courses (typically four times daily dosing for 3 to 5 days). Alternative oral agents may have been possible in some cases, but 31/75 patients in this cohort had no other oral alternative. In these instances, fosfomycin saves the cost, risks and time taken to provide intravenous therapy.

The issue of selection and spread of resistance is complex [6, 16, 21, 30, 31]. Notably, in this study, the post-treatment isolate in 5/19 cases of microbiological failure was fosfomycin-resistant, all of which were non-*E. coli* Enterobacteriaceae. Fosfomycin resistance can be caused by a number of different mechanisms, including chromosomal mutations in genes encoding membrane transport systems or regulators of these transporters (*uhpA*, *uhpT*, *glpT*, *ptsI*), modification of murA (the drug’s target of action), or the presence of catalytic enzymes encoded by *fos* genes, some of which can be transferred on mobile genetic elements [21, 30, 31]. A recent analysis reports a fosfomycin resistance rate of 0.5 % in community-acquired *E. coli* UTI in women in the UK [24]; it is unsurprising that our figure is higher (1 %) as this includes a hospital population at more risk of MDR organisms.

Data from both in vitro and clinical studies suggest that the emergence of resistance during treatment is higher for *Pseudomonas aeruginosa*, *Proteus*, *Klebsiella* and *Enterobacter* spp., as opposed to *E. coli*, [21, 27] consistent with what we observed here, and suggesting that fosfomycin should be used with caution in infections caused by these organisms.

In this current study, multivariate analysis is somewhat limited by small patient numbers and by missing data (due to the retrospective approach). Furthermore, the selection of variables entered into a multivariate analysis is characteristically based on those which reach statistical significance at univariate level; in this case only *Klebsiella* infection reached this threshold. Nevertheless, entering factors that could reasonably be hypothesized to be predictive of treatment failure into multivariate analysis, Klebsiella still emerged as significantly associated with failure (*p* = 0.04). A small study from Hong Kong also points to a higher rates of failure in *Klebsiella* compared to *E. coli* UTI [27]. The benefits of more widespread use of fosfomycin therefore have to be balanced against the dual risks of increasing selection pressures for resistance, and these preliminary data suggesting that it may be less efficacious against Klebsiella infection, irrespective of in vitro susceptibility.

There are several caveats and limitations to this study. Although our cohort is larger than previous equivalent studies (Additional file 1: Table S1), our power to derive statistically significant findings is still limited by small numbers of patients in each individual subgroup (highlighted by the breakdown of characteristics presented in Tables 1, 2, 4 and 5). Certain data (e.g. co-morbidities and involvement of the microbiology team) may have been under-reported in the EPR. We did not have access to complete paper records for the full cohort, so we have not been able to provide a detailed description of patient symptoms at the outset of treatment. Thus we have not been able to discriminate between asymptomatic bacteriuria and symptomatic UTI, or to identify features to distinguish between upper and lower UTI.

The retrospective study design also made assessment of clinical and microbiological end-points challenging, and made it impossible to justify why fosfomycin was chosen for treatment in each individual case. A related problem is the tertiary referral nature of our centre, which means that relevant culture data may have been generated elsewhere, and this may have led to missing culture data at the outset of treatment, and a potential over-estimation of cases of functional cure. Further prospective studies are undoubtedly warranted to scrutinize prescribing and outcomes in larger cohorts, to collect more complete and robust clinical data and to provide a more reliable assessment of final outcome, including seeking information from primary care regarding previous or subsequent courses of antibacterial therapy. Those wishing to apply our conclusions to their own centres should consider carefully the extent to which the cohort and epidemiology described here is comparable to their own patient populations.

Nevertheless, as a consequence of the data presented here, we have reviewed our local approach to fosfomycin prescribing, and have produced new guidelines with a view to making our own use of fosfomycin more rational and consistent. These include the following recommendations:

i. Use of an alternative oral agent, particularly nitrofurantoin, should be considered first; fosfomycin should only be used if other oral options are contraindicated on the grounds of in vitro susceptibility data or clinical factors (drug allergy, pregnancy, renal impairment, previous treatment failure);
ii. Fosfomycin may be used in patients with UTI caused by non-*E. coli* Enterobacteriaceae, but only after careful consideration, particularly considering the potential risk of treatment failure for infection caused by *Klebsiella* spp.;
iii. The number of doses of fosfomycin treatment should be carefully considered: gender-based dosing is suggested by NICE, although this approach is not uniform. Additional doses may be used for patients in whom eradication of infection is difficult.
iv. Underlying medical or surgical risk factors should not be considered a contra-indication to treatment with fosfomycin, and this preliminary evidence suggests that prolonged courses are likely to be safe and well tolerated; however, such patients need to be kept under close expert review.
v. The optimum way to ensure that all of these factors are considered is to reinforce the recommendation that all individuals receiving oral fosfomycin are discussed with the infectious diseases/microbiology team. We have added fosfomycin to the electronic guidelines available to clinicians via the ‘Microguide’ app (http://microguide.eu/).
vi. We have implemented a protocol for primary care physicians to access fosfomycin for patients in the community, following telephone or email approval from a hospital microbiologist.

## Conclusions

Overall, our findings underline the important role of fosfomycin in the antibacterial armamentarium for treatment of UTI. At present, the evidence of benefit is strongest for *E. coli* infections, and careful stewardship is important to reduce the risk of selection of antimicrobial resistance. This study also provides preliminary data to suggest that prolonged courses of this drug are safe and can be effective in suppressing or eradicating multi-resistant organisms even in immunosuppressed patients and in the setting of complex, abnormal renal tract anatomy.

## Additional files

Additional file 1: Spreadsheet reporting 75 unique patients treated with fosfomycin for urinary tract infections. This excel file contains anonymised details of 75 unique adult patients treated with fosfomycin for urinary tract infections at Oxford University Hospitals NHS Foundation Trust from March 2013 through June 2015. Our original analysis also included patient sex, but this has been removed from the suppl. data file in order to protect anonymity. (XLSX 24 kb)
Additional file 2: Tabulated summary of studies that have informed current NICE guidelines for fosfomycin use in UTI. This table contains a brief summary of four studies from Europe/North America that are referenced by current NICE guidelines regarding the use of oral fosfomycin for treatment of UTI, including date of study, cohort location, study design and cure data. (DOCX 88 kb)

## Abbreviations

BSAC: British Society for Antimicrobial Chemotherapy
*E. coli*: *Escherichia coli*
eGFR: Estimated glomerular filtration rate
EPR: Electronic patient record
ESBL: Extended spectrum beta-lactamase
EUCAST: European Committee for Antimicrobial Susceptibility Testing
MIC: Minimum inhibitory concentration
NICE: National Institute for Health and Care Excellence
PHE: Public Health England
UTI: Urinary tract infection
