# Supplementary material for: Oral fosfomycin for treatment of urinary tract infection: a retrospective cohort study
Source: BMC Infect Dis. 2016 Oct 11;16:556. doi: 10.1186/s12879-016-1888-1 (PMC5057270; doi:10.1186/s12879-016-1888-1)
Supplement: Additional file 2: — Tabulated summary of studies that have informed current NICE guidelines for fosfomycin use in UTI. This table contains a brief summary of four studies from Europe/North America that are referenced by current NICE guidelines regarding the use of oral fosfomycin for treatment of UTI, including date of study, cohort location, study design and cure data. (DOCX 88 kb) [file 12879_2016_1888_MOESM2_ESM.docx]

**Additional file 2: TABLE S2: Summary of four studies used to inform NICE guidelines^16^ for the treatment of UTI with oral fosfomycin**

| **Author, date of publication** | **Dates study conducted** | **Location of study** | **Number of subjects treated with fosfomycin** | **Study design** | **Cure data** |
| --- | --- | --- | --- | --- | --- |
| Pullukcu *et al*., 2007 | 2004-2006 | Turkey | 52 | Retrospective review of fosfomycin use for adults with uncomplicated ESBL *E. coli* lower UTI | Clinical cure in 49/52 (94%); microbiological cure in 41/52 (79%). |
| Rodriguez-Bano *et al*., 2008 | 2002-2003 | Spain | 65 | Case control study of fosfomycin vs co-amoxiclav in outpatients with cystitis caused by *E. coli*  (+/-ESBL) | Clinical cure in 26/28 (93%); comparable outcome between  the two agents |
| Senol *et al*., 2010 | 2005-2006 | Turkey | 27 | Observational prospective study comparing carbapenem vs. fosfomycin for ESBL-positive  *E. coli* UTI | Clinical cure in 21/27 (78%); microbiological cure in 16/27 (59%). No significant difference in outcomes between carbapenem and fosfomycin treatment. |
| Neuner *et al*., 2012 | 2006-2010 | USA | 41 | Retrospective review of fosfomycin use for MDR UTI in women (hospital in-patients, various pathogens) | Microbiological cure in 24/41 (59%); solid organ transplant and ureteric stents predicted failure. |
